# Supplementary material for: Comparative Study of Three Mixing Methods in Fusion Technique for Determining Major and Minor Elements Using Wavelength Dispersive X-ray Fluorescence Spectroscopy
Source: Sensors (Basel). 2020 Sep 17;20(18):5325. doi: 10.3390/s20185325 (PMC7570679; doi:10.3390/s20185325)
Supplement: Supplementary file 1 [file sensors-20-05325-s001.pdf]

Supplementary Materials

Comparative study of three mixing methods in fusion technique for determining major and minor elements using wavelength dispersive X-ray fluorescence spectroscopy

Dan-ping Zhang [1,2,3], Ding-shuai Xue [1,*], Yan-hong Liu [1], Bo Wan [1], Qian Guo [1], and Ju-jie Guo [1]

[1] State Key Laboratory of Lithospheric Evolution, Institute of Geology and Geophysics, Chinese Academy of Sciences, Beijing 100029, China; zdanp@mail.iggcas.ac.cn; liuyanhong@mail.iggcas.ac.cn (Y.-h. L.); wanbo@mail.iggcas.ac.cn (B. W.); guoqian@mail.iggcas.ac.cn (Q. G.); guojujie@mail.iggcas.ac.cn (J.-j. G.)

[2] Innovation Academy for Earth Science, Chinese Academy of Sciences, Beijing, 100029, China

[3] Institute of Geology and Geophysics, Chinese Academy of Sciences, Beijing, 100029, China

* *Correspondence: xuedingshuai@mail.iggcas.ac.cn; Tel.: +86-010-82998487*

Table S1. Details of the certified reference materials (CRMs).

Source	CRMs used for the calibration		CRMs used for the validation
IGGE[a]	GBW07101 ultramafic rock<br>GBW07102 ultramafic rock<br>GBW07103 granite<br>GBW07104 andesite<br>GBW07105 basalt<br>GBW07106 sandstone<br>GBW07107 shale<br>GBW07108 carbonate<br>GBW07109 syenite<br>GBW07110 trachyte<br>GBW07111 diorite	GBW07112 gabbro<br>GBW07113 rhyolite<br>GBW07114 dolomite<br>GBW07120 limestone<br>GBW07121 granite-gneiss<br>GBW07122 hornblendite<br>GBW07123 diabase<br>GBW07124 kimberlite<br>GBW07125 pegmatite<br>GBW07212 phosphorite<br>GBW07312 stream sediment<br>GBW07406 soil	
GSJ[b]	JG-1 granite<br>JG-3 granodiorite<br>JA-1 andesite<br>JA-2 andesite<br>JB-1b basalt<br>JB-2 basalt<br>JB-3 basalt<br>JGb-1 gabbro	JR-1 rhyolite<br>JP-1 peridotite<br>JF-1 feldspar	JG-2 Granite<br>JG-1a Granodiorite<br>JA-3 Andesite<br>JB-2a Basalt<br>JB-3a Basalt<br>JH-1 Hornblendite<br>JGb-2 Gabbro<br>JR-2 Rhyolite
USGS[c]	AGV-2 andesite<br>BIR-1a basalt<br>QLO-1a quartz latite<br>SBC-1 shale<br>COQ-1 carbonatite	STM-2 syenite<br>DNC-1a diabase<br>DTS-2B dunite<br>SDC-1 mica schist	GSP-2 Granodiorite<br>BCR-2 Basalt<br>BHVO-2 Basalt<br>W-2 Diabase<br>RGM-2 Rhyolite
ANRT[d]	GA granite<br>Mica-Fe biotite<br>Mica-Mg phlogopite	DT-N gabbro<br>FK-N feldspar	
CRPG[e]	DR-N diorite<br>GS-N granite<br>UB-N serpentinite		GH Granite
IWG-GIT[f]	MA-N granite<br>BE-N basalt<br>AL-I albite		AC-E Granite<br>PM-S Microgabbro<br>WS-E Dolerite<br>AN-G Anorthosite
CGL[g]			MGL-OShBO Granite

[a]Institute of Geophysical and Geochemical Exploration. China. [b]Geological Survey of Japan. AIST. [c]United States Geological Survey. Denver, CO, USA. [d]Association Nationale de la Recherche Technique. [e]Centre de Recherches Pétrographiques et Géochimiques. [f]International Working Group "Analytical Standards of Minerals, Ores, and Rocks" - Groupe International de Travail "Etalons analytiques des mineraux, minerals et roches". [g]The Central Geological Laboratory of Mongolia.

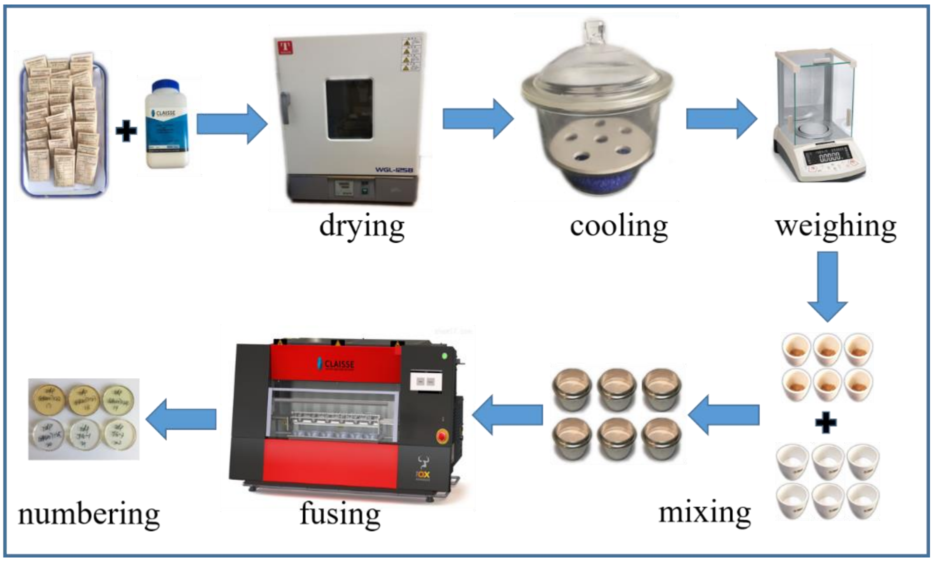

Figure S1. Fused glass disc preparation procedure

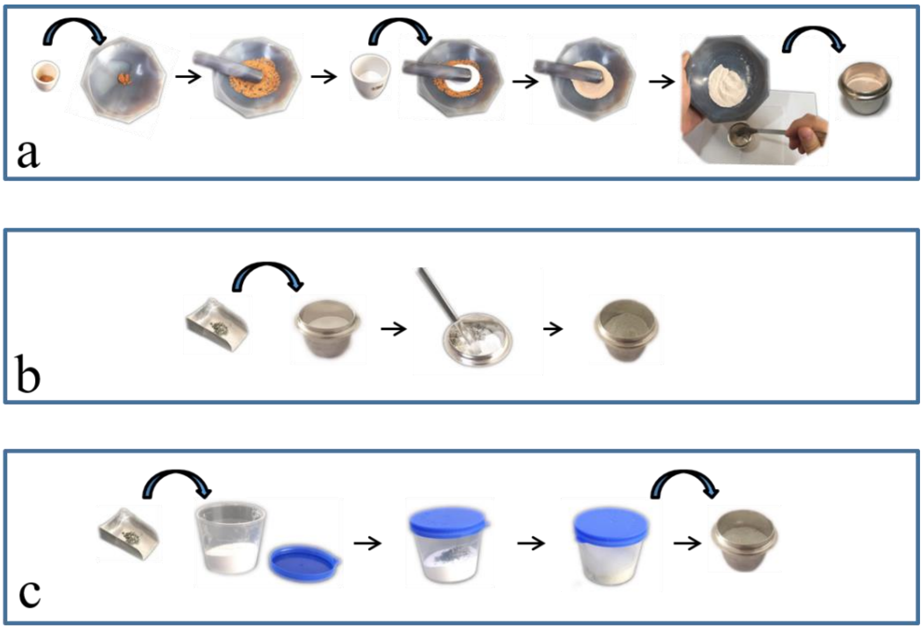

Figure S2. Procedures for mixing the samples and flux by (a) grinding, (b) stirring, and (c) shaking.

Table S2. Results of the major and minor element determination by X-ray fluorescence (XRF) analysis of the samples prepared by the three preparation procedures.

JG-2 (granite)

Major	This work			Literature	Minor	This work			Literature
	$X_{GR} \pm SD_{GR}$	$X_{ST} \pm SD_{ST}$	$X_{SH} \pm SD_{SH}$	$X_{lit} \pm SD_{lit}$		$X_{GR} \pm SD_{GR}$	$X_{ST} \pm SD_{ST}$	$X_{SH} \pm SD_{SH}$	$X_{lit} \pm SD_{lit}$
SiO_2	76.98 ± 0.06	76.66 ± 0.09	77.02 ± 0.08	76.83 ± 0.57	Cr	*5.0 ± 8.4*	*6.1 ± 2.4*	*7.0 ± 8.6*	6.37 ± 2.09
TiO_2	0.051 ± 0.002	0.047 ± 0.001	0.048 ± 0	0.044 ± 0.009	Cu	*9.2 ± 12.1*	*16.9 ± 3.2*	*14.2 ± 2.7*	0.49 ± 0.12
Al_2O_3	12.44 ± 0.05	12.40 ± 0.03	12.40 ± 0.01	12.47 ± 0.32	Ba	70.0 ± 35.4	76.8 ± 26.2	55.2 ± 8.1	81.0 ± 23.5
TFe_2O_3	0.98 ± 0.02	0.97 ± 0.01	0.99 ± 0.03	0.97 ± 0.06	Ni	*5.0 ± 12.9*	*-0.3 ± 1.9*	*-2.1 ± 1.8*	4.35 ± 3.07
MnO	0.020 ± 0	0.019 ± 0	0.018 ± 0	0.016 ± 0.003	Sr	*-27.1 ± 0.1*	*-24.4 ± 1.7*	*-13.1 ± 1.9*	17.9 ± 3.5
MgO	0.053 ± 0.006	0.050 ± 0	0.050 ± 0	0.037 ± 0.012	V	*1.0 ± 3.7*	*5.7 ± 4.2*	*-4.3 ± 5.3*	3.78 ± 1.58
CaO	0.69 ± 0.01	0.70 ± 0	0.70 ± 0	0.70 ± 0.05	Zr	104.0 ± 2.6	105.5 ± 1.6	90.9 ± 2.0	97.6 ± 10.8
Na_2O	3.57 ± 0.14	3.51 ± 0.01	3.55 ± 0.01	3.54 ± 0.11	Zn	7.6 ± 15.0	*-0.7 ± 1.3*	*0.8 ± 1.9*	13.6 ± 2.4
K_2O	4.69 ± 0.01	4.68 ± 0.01	4.72 ± 0.01	4.71 ± 0.09					
P_2O_5	0.012 ± 0.001	0.008 ± 0	0.011 ± 0.001	0.002 ± 0					
LOI	0.41 ± 0.03	0.41 ± 0.03	0.41 ± 0.03	NRV					
Total	99.90	99.45	99.91	NC					

JG-1a (granodiorite)

Major	This work			Literature	Minor	This work			Literature
	$X_{GR} \pm SD_{GR}$	$X_{ST} \pm SD_{ST}$	$X_{SH} \pm SD_{SH}$	$X_{lit} \pm SD_{lit}$		$X_{GR} \pm SD_{GR}$	$X_{ST} \pm SD_{ST}$	$X_{SH} \pm SD_{SH}$	$X_{lit} \pm SD_{lit}$
SiO_2	72.40 ± 0.13	72.34 ± 0.02	72.45 ± 0.10	72.30 ± 0.51	Cr	14.2 ± 2.4	14.1 ± 5.3	15.8 ± 4.6	17.6 ± 4.4
TiO_2	0.240 ± 0.006	0.243 ± 0.002	0.244 ± 0.001	0.25 ± 0.03	Cu	*3.8 ± 3.0*	*14.5 ± 3.2*	*12.8 ± 1.2*	1.67 ± 0.59
Al_2O_3	14.15 ± 0.05	14.08 ± 0.03	14.13 ± 0.02	14.30 ± 0.41	Ba	478.5 ± 15.8	469.8 ± 18.9	486.3 ± 19.1	470 ± 38
TFe_2O_3	1.96 ± 0.01	1.95 ± 0.01	1.94 ± 0.01	2.00 ± 0.10	Ni	*-0.9 ± 0.8*	*1.5 ± 1.4*	*8.8 ± 3.1*	6.91 ± 1.90
MnO	0.060 ± 0	0.059 ± 0	0.058 ± 0	0.057 ± 0.007	Sr	183.0 ± 0.7	187.8 ± 0.5	195.4 ± 0.7	187 ± 12
MgO	0.69 ± 0.01	0.70 ± 0	0.70 ± 0.01	0.69 ± 0.07	V	21.7 ± 1.0	22.4 ± 5.7	26.2 ± 1.1	22.7 ± 3.8
CaO	2.15 ± 0.02	2.16 ± 0.01	2.16 ± 0.01	2.13 ± 0.08	Zr	115.6 ± 2.9	117.0 ± 1.8	117.7 ± 9.4	118 ± 13
Na_2O	3.37 ± 0.04	3.37 ± 0.01	3.41 ± 0	3.39 ± 0.13	Zn	29.1 ± 3.8	35.4 ± 1.2	33.5 ± 2.8	36.5 ± 2.2
K_2O	3.96 ± 0.01	3.97 ± 0	3.98 ± 0.01	3.96 ± 0.16					
P_2O_5	0.083 ± 0.001	0.083 ± 0.001	0.084 ± 0.001	0.083 ± 0.009					
LOI	0.68 ± 0.05	0.68 ± 0.05	0.68 ± 0.05	NRV					

Total	99.75	99.63	99.83	NC					
JA-3 (andesite)									
Major	This work			Literature	Minor	This work			Literature
	$X_{GR} \pm SD_{GR}$	$X_{ST} \pm SD_{ST}$	$X_{SH} \pm SD_{SH}$	$X_{lit} \pm SD_{lit}$		$X_{GR} \pm SD_{GR}$	$X_{ST} \pm SD_{ST}$	$X_{SH} \pm SD_{SH}$	$X_{lit} \pm SD_{lit}$
SiO_2	61.87 ± 0.26	62.04 ± 0.10	62.10 ± 0.05	62.27 ± 0.34	Cr	64.9 ± 8.0	65.1 ± 4.7	64.9 ± 1.2	66.2 ± 10.0
TiO_2	0.650 ± 0.013	0.668 ± 0.004	0.675 ± 0.001	0.70 ± 0.06	Cu	43.5 ± 11.7	44.7 ± 1.2	45.0 ± 0.2	43.4 ± 3.9
Al_2O_3	15.62 ± 0.08	15.65 ± 0.06	15.69 ± 0.02	15.56 ± 0.20	Ba	323.7 ± 21.8	306.9 ± 13.0	327.1 ± 20.8	323 ± 28
TFe_2O_3	6.50 ± 0.02	6.49 ± 0.01	6.49 ± 0.01	6.60 ± 0.18	Ni	42.0 ± 25.0	24.9 ± 1.4	27.7 ± 2.5	32.2 ± 6.6
MnO	0.103 ± 0.006	0.103 ± 0	0.103 ± 0.001	0.104 ± 0.010	Sr	280.7 ± 4.5	294.0 ± 3.6	297.3 ± 2.5	287 ± 18
MgO	3.66 ± 0.02	3.67 ± 0.01	3.69 ± 0.01	3.72 ± 0.09	V	158.3 ± 3.6	160.2 ± 4.2	168.2 ± 3.1	169 ± 16
CaO	6.33 ± 0.01	6.34 ± 0.01	6.34 ± 0.02	6.24 ± 0.16	Zr	108.4 ± 0.8	106.8 ± 2.7	105.3 ± 0.8	118 ± 9.6
Na_2O	3.16 ± 0.05	3.18 ± 0.01	3.19 ± 0.01	3.19 ± 0.10	Zn	69.2 ± 8.1	67.3 ± 2.4	70.6 ± 3.4	67.7 ± 5.2
K_2O	1.37 ± 0.01	1.37 ± 0	1.38 ± 0	1.41 ± 0.06					
P_2O_5	0.112 ± 0.001	0.116 ± 0.001	0.114 ± 0.001	0.116 ± 0.019					
LOI	0.18 ± 0.06	0.18 ± 0.06	0.18 ± 0.06	NRV					
Total	99.54	99.79	99.95	NC					
JB-2a (basalt)									
Major	This work			Literature	Minor	This work			Literature
	$X_{GR} \pm SD_{GR}$	$X_{ST} \pm SD_{ST}$	$X_{SH} \pm SD_{SH}$	$X_{lit} \pm SD_{lit}$		$X_{GR} \pm SD_{GR}$	$X_{ST} \pm SD_{ST}$	$X_{SH} \pm SD_{SH}$	$X_{lit} \pm SD_{lit}$
SiO_2	53.28 ± 0.13	53.16 ± 0.02	53.30 ± 0.06	53.22	Cr	24.9 ± 3.5	30.0 ± 5.1	29.5 ± 5.7	28
TiO_2	1.106 ± 0.023	1.139 ± 0.006	1.149 ± 0.003	1.18	Cu	201.3 ± 2.2	207.7 ± 2.6	205.6 ± 3.3	269
Al_2O_3	14.67 ± 0.05	14.66 ± 0.02	14.72 ± 0.02	14.67	Ba	226.6 ± 24.4	217.4 ± 19.0	219.9 ± 16.1	219
TFe_2O_3	14.13 ± 0.03	14.08 ± 0.01	14.10 ± 0.03	14.18	Ni	11.2 ± 5.5	8.9 ± 3.5	14.1 ± 0.6	14.5
MnO	0.217 ± 0.006	0.212 ± 0.001	0.211 ± 0.001	0.214	Sr	140.0 ± 2.9	150.0 ± 1.0	147.2 ± 1.8	179
MgO	4.56 ± 0.01	4.54 ± 0.01	4.57 ± 0.02	4.58	V	558.3 ± 4.9	564.6 ± 6.0	579.9 ± 7.7	574
CaO	9.75 ± 0.03	9.72 ± 0.02	9.75 ± 0.02	9.79	Zr	58.5 ± 0.7	54.2 ± 1.3	52.1 ± 0.7	61.8
Na_2O	2.03 ± 0.02	2.03 ± 0.01	2.05 ± 0.01	2.03	Zn	96.8 ± 3.1	100.8 ± 1.4	105.6 ± 4.1	107
K_2O	0.41 ± 0	0.41 ± 0	0.41 ± 0	0.41					
P_2O_5	0.096 ± 0.001	0.097 ± 0.001	0.098 ± 0.001	0.095					
LOI	-0.56 ± 0.08	-0.56 ± 0.08	-0.56 ± 0.08	NAV					

Total	99.69	99.48	99.81	NC					

JB-3a (basalt)									
Major	This work			Literature	Minor	This work			Literature
	$X_{GR} \pm SD_{GR}$	$X_{ST} \pm SD_{ST}$	$X_{SH} \pm SD_{SH}$	$X_{lit} \pm SD_{lit}$		$X_{GR} \pm SD_{GR}$	$X_{ST} \pm SD_{ST}$	$X_{SH} \pm SD_{SH}$	$X_{lit} \pm SD_{lit}$
SiO_2	50.90 ± 0.06	50.88 ± 0.04	50.96 ± 0.17	50.87	Cr	52.8 ± 2.2	56.8 ± 4.4	54.8 ± 5.1	57
TiO_2	1.346 ± 0.025	1.383 ± 0.002	1.401 ± 0.005	1.44	Cu	138.6 ± 4.4	151.6 ± 2.1	150.8 ± 1.2	195
Al_2O_3	17.27 ± 0.07	17.29 ± 0.02	17.29 ± 0.02	17.16	Ba	243.1 ± 10.5	243.1 ± 15.7	244.2 ± 12.0	244
TFe_2O_3	11.73 ± 0.01	11.66 ± 0.02	11.69 ± 0.01	11.83	Ni	29.3 ± 2.1	34.0 ± 4.0	34.1 ± 2.9	39
MnO	0.180 ± 0	0.174 ± 0	0.174 ± 0	0.179	Sr	389.1 ± 7.0	398.0 ± 4.6	405.8 ± 3.1	404
MgO	5.10 ± 0.03	5.10 ± 0.01	5.13 ± 0.01	5.17	V	364.0 ± 6.0	383.7 ± 2.8	385.6 ± 3.9	375
CaO	9.64 ± 0.02	9.63 ± 0.01	9.65 ± 0.03	9.75	Zr	95.7 ± 0.2	90.7 ± 0.2	95.4 ± 0.3	100
Na_2O	2.71 ± 0.02	2.74 ± 0.01	2.75 ± 0.01	2.74	Zn	98.4 ± 8.0	104.3 ± 2.1	105.7 ± 1.7	104
K_2O	0.75 ± 0	0.75 ± 0	0.75 ± 0	0.78					
P_2O_5	0.282 ± 0.002	0.290 ± 0.002	0.283 ± 0.001	0.291					
LOI	-0.38 ± 0	-0.38 ± 0	-0.38 ± 0	NAV					
Total	99.53	99.52	99.70	NC					

JH-1 (hornblendite)									
Major	This work			Literature	Minor	This work			Literature
	$X_{GR} \pm SD_{GR}$	$X_{ST} \pm SD_{ST}$	$X_{SH} \pm SD_{SH}$	$X_{lit} \pm SD_{lit}$		$X_{GR} \pm SD_{GR}$	$X_{ST} \pm SD_{ST}$	$X_{SH} \pm SD_{SH}$	$X_{lit} \pm SD_{lit}$
SiO_2	47.91 ± 0.12	47.81 ± 0.06	47.91 ± 0.08	48.18 ± 0.695	Cr	596.8 ± 16.2	593.9 ± 7.9	599.5 ± 2.0	616 ± 54.4
TiO_2	0.648 ± 0.013	0.658 ± 0.005	0.662 ± 0.003	0.665 ± 0.017	Cu	*3.7 ± 10.1*	*10.6 ± 3.8*	*8.8 ± 1.1*	8.61 ± 1.33
Al_2O_3	5.68 ± 0.05	5.67 ± 0.01	5.68 ± 0.01	5.657 ± 0.248	Ba	130.9 ± 15.3	108.6 ± 13.7	132.4 ± 7.7	106 ± 14.2
TFe_2O_3	10.17 ± 0.05	10.09 ± 0.01	10.12 ± 0.01	10.27 ± 0.164	Ni	64.4 ± 43.4	51.3 ± 2.4	52.5 ± 1.5	58.2 ± 8.19
MnO	0.180 ± 0	0.177 ± 0	0.177 ± 0	0.190 ± 0.0084	Sr	122.0 ± 3.9	130.4 ± 0.5	124.6 ± 1.2	153 ± 5.48
MgO	16.50 ± 0.08	16.49 ± 0.01	16.59 ± 0.03	16.73 ± 0.593	V	220.3 ± 4.4	224.7 ± 4.5	236.8 ± 5.9	228 ± 7.86
CaO	14.63 ± 0.03	14.54 ± 0.01	14.57 ± 0.04	15.02 ± 0.20	Zr	52.9 ± 1.5	50.6 ± 1.2	41.8 ± 0.4	48.3 ± 5.30
Na_2O	0.70 ± 0.02	0.69 ± 0	0.70 ± 0.01	0.707 ± 0.027	Zn	57.8 ± 16.5	51.0 ± 3.4	57.0 ± 1.8	61.8 ± 6.0
K_2O	0.52 ± 0	0.53 ± 0	0.53 ± 0	0.526 ± 0.021					
P_2O_5	0.100 ± 0.001	0.100 ± 0.001	0.100 ± 0	0.0991 ± 0.0102					
LOI	2.13 ± 0.10	2.13 ± 0.10	2.13 ± 0.10	1.780 ± 0.324					

Total	99.17	98.90	99.16	99.82					
JGb-2 (gabbro)									
Major	This work			Literature	Minor	This work			Literature
	$X_{GR} \pm SD_{GR}$	$X_{ST} \pm SD_{ST}$	$X_{SH} \pm SD_{SH}$	$X_{lit} \pm SD_{lit}$		$X_{GR} \pm SD_{GR}$	$X_{ST} \pm SD_{ST}$	$X_{SH} \pm SD_{SH}$	$X_{lit} \pm SD_{lit}$
SiO_2	46.28 ± 0.13	46.11 ± 0.13	46.19 ± 0.07	46.47 ± 0.365	Cr	117.8 ± 6.2	119.7 ± 3.4	116.1 ± 2.4	125 ± 4.91
TiO_2	0.546 ± 0.011	0.552 ± 0.003	0.559 ± 0.001	0.561 ± 0.016	Cu	2.5 ± 1.5	14.4 ± 2.3	14.2 ± 0.8	11.4 ± 1.79
Al_2O_3	23.51 ± 0.09	23.45 ± 0.03	23.49 ± 0.02	23.48 ± 0.272	Ba	*27.5 ± 7.9*	*37.4 ± 11.7*	*11.5 ± 18.4*	36.5 ± 7.75
TFe_2O_3	6.68 ± 0.02	6.62 ± 0.01	6.64 ± 0.01	6.691 ± 0.137	Ni	28.7 ± 43.2	11.2 ± 1.4	8.6 ± 3.5	13.6 ± 1.90
MnO	0.130 ± 0	0.123 ± 0.001	0.123 ± 0	0.126 ± 0.010	Sr	450.7 ± 1.4	458.9 ± 2.3	462.7 ± 1.1	438 ± 12.3
MgO	6.05 ± 0.03	6.06 ± 0.03	6.11 ± 0.02	6.183 ± 0.119	V	165.6 ± 6.9	169.6 ± 1.3	175.4 ± 5.4	174 ± 5.28
CaO	13.73 ± 0.04	13.66 ± 0.03	13.7 ± 0.02	14.10 ± 0.113	Zr	12.8 ± 1.5	11.2 ± 0.9	35.9 ± 0.6	11.6 ± 4.93
Na_2O	0.94 ± 0.04	0.92 ± 0	0.95 ± 0.01	0.921 ± 0.027	Zn	34.6 ± 0.6	40.7 ± 2.9	42.0 ± 1.8	48.5 ± 5.47
K_2O	0.060 ± 0	0.056 ± 0	0.062 ± 0	0.059 ± 0.0073					
P_2O_5	0.015 ± 0.001	0.012 ± 0	0.016 ± 0.001	0.017 ± 0.0080					
LOI	1.36 ± 0.03	1.36 ± 0.03	1.36 ± 0.03	1.357 ± 0.154					
Total	99.31	98.94	99.20	99.97					
JR-2 (rhyolite)									
Major	This work			Literature	Minor	This work			Literature
	$X_{GR} \pm SD_{GR}$	$X_{ST} \pm SD_{ST}$	$X_{SH} \pm SD_{SH}$	$X_{lit} \pm SD_{lit}$		$X_{GR} \pm SD_{GR}$	$X_{ST} \pm SD_{ST}$	$X_{SH} \pm SD_{SH}$	$X_{lit} \pm SD_{lit}$
SiO_2	75.28 ± 0.15	75.32 ± 0.18	75.56 ± 0.11	75.69 ± 0.37	Cr	*2.5 ± 3.2*	*6.9 ± 3.4*	*2.7 ± 3.5*	3.10 ± 1.18
TiO_2	0.066 ± 0.003	0.063 ± 0.003	0.063 ± 0.001	0.07 ± 0.01	Cu	*5.5 ± 2.0*	*17.2 ± 1.2*	*15.2 ± 1.5*	1.36 ± 0.47
Al_2O_3	12.54 ± 0.01	12.52 ± 0.04	12.61 ± 0.01	12.72 ± 0.28	Ba	*12.8 ± 11.6*	*39.2 ± 17.7*	*19.7 ± 18.3*	39.5 ± 17.2
TFe_2O_3	0.77 ± 0.01	0.69 ± 0.01	0.71 ± 0	0.77 ± 0.06	Ni	*-3.5 ± 2.8*	*-0.3 ± 4.6*	*-0.5 ± 1.5*	1.99 ± 1.59
MnO	0.11 ± 0	0.107 ± 0	0.107 ± 0.001	0.112 ± 0.007	Sr	*-37.8 ± 0.7*	*-35.7 ± 1.0*	*-24.0 ± 2.2*	8.11 ± 2.31
MgO	0.06 ± 0.01	0.05 ± 0	0.05 ± 0.01	0.04 ± 0.01	V	*3.3 ± 5.5*	*2.3 ± 6.1*	*6.4 ± 6.9*	3.00 ± 1.76
CaO	0.52 ± 0.01	0.53 ± 0.01	0.52 ± 0.01	0.50 ± 0.06	Zr	92.8 ± 0.9	93.0 ± 0.5	80.4 ± 0.5	96.3 ± 6.4
Na_2O	3.94 ± 0.02	3.98 ± 0.01	4 ± 0.01	3.99 ± 0.18	Zn	14.4 ± 4.6	20.9 ± 1.7	22.0 ± 1.8	27.8 ± 2.7
K_2O	4.44 ± 0.01	4.45 ± 0.01	4.46 ± 0	4.45 ± 0.12					
P_2O_5	0.012 ± 0	0.008 ± 0.001	0.012 ± 0.001	0.012 ± 0.005					
LOI	1.95 ± 0.03	1.95 ± 0.03	1.95 ± 0.03	NRV					

Total	99.69	99.66	100.05	NC					
GSP-2 (granodiorite)									
Major	This work			Literature	Minor	This work			Literature
	$X_{GR} \pm SD_{GR}$	$X_{ST} \pm SD_{ST}$	$X_{SH} \pm SD_{SH}$	$X_{lit} \pm SD_{lit}$		$X_{GR} \pm SD_{GR}$	$X_{ST} \pm SD_{ST}$	$X_{SH} \pm SD_{SH}$	$X_{lit} \pm SD_{lit}$
SiO_2	66.41 ± 0.10	66.40 ± 0.18	66.82 ± 0.12	66.60 ± 0.8	Cr	11.4 ± 2.3	20.5 ± 3.7	15.4 ± 4.1	20 ± 6
TiO_2	0.640 ± 0.013	0.661 ± 0.002	0.665 ± 0.002	0.66 ± 0.02	Cu	43.3 ± 6.5	49.7 ± 0.6	52.1 ± 5.5	43 ± 4
Al_2O_3	14.76 ± 0.04	14.76 ± 0.05	14.9 ± 0.04	14.90 ± 0.2	Ba	1331.3 ± 12.2	1331.0 ± 26.2	1324.7 ± 23.0	1340 ± 44
TFe_2O_3	4.87 ± 0.02	4.87 ± 0.01	4.88 ± 0.01	4.90 ± 0.16	Ni	10.8 ± 1.7	13.0 ± 1.6	15.0 ± 2.1	17 ± 2
MnO	0.040 ± 0	0.041 ± 0	0.042 ± 0	0.041 ± 0.003	Sr	236.8 ± 2.2	247.5 ± 2.8	251.7 ± 4.5	240 ± 10
MgO	0.95 ± 0.01	0.96 ± 0.01	0.97 ± 0.01	0.96 ± 0.03	V	45.8 ± 4.4	47.2 ± 3.4	43.2 ± 6.0	52 ± 4
CaO	2.08 ± 0.02	2.10 ± 0.01	2.10 ± 0.01	2.10 ± 0.06	Zr	529.1 ± 7.4	538.1 ± 9.7	493.7 ± 8.9	550 ± 30
Na_2O	2.77 ± 0.01	2.79 ± 0	2.81 ± 0.01	2.78 ± 0.09	Zn	124.1 ± 4.1	135.7 ± 0.5	137.6 ± 1.4	120 ± 10
K_2O	5.36 ± 0.01	5.38 ± 0.02	5.40 ± 0	5.38 ± 0.14					
P_2O_5	0.281 ± 0.003	0.289 ± 0.002	0.285 ± 0.001	0.29 ± 0.02					
LOI	1.12 ± 0.52	1.12 ± 0.52	1.12 ± 0.52	NRV					
Total	99.27	99.36	99.99	NC					
BCR-2 (basalt)									
Major	This work			Literature	Minor	This work			Literature
	$X_{GR} \pm SD_{GR}$	$X_{ST} \pm SD_{ST}$	$X_{SH} \pm SD_{SH}$	$X_{lit} \pm SD_{lit}$		$X_{GR} \pm SD_{GR}$	$X_{ST} \pm SD_{ST}$	$X_{SH} \pm SD_{SH}$	$X_{lit} \pm SD_{lit}$
SiO_2	54.16 ± 0.02	54.19 ± 0.12	54.17 ± 0.09	54.10 ± 0.8	Cr	14.7 ± 0.7	17.8 ± 7.9	18.0 ± 1.2	18 ± 2
TiO_2	2.338 ± 0.005	2.310 ± 0.005	2.293 ± 0.006	2.26 ± 0.05	Cu	5.2 ± 1.0	16.4 ± 1.2	13.8 ± 2.3	19 ± 2
Al_2O_3	13.37 ± 0.03	13.4 ± 0.03	13.39 ± 0.02	13.50 ± 0.2	Ba	661.6 ± 22.8	679.7 ± 22.5	679.1 ± 20.3	683 ± 28
TFe_2O_3	13.82 ± 0.02	13.78 ± 0.03	13.76 ± 0.02	13.80 ± 0.2	Ni	12.1 ± 2.5	11.2 ± 0.7	12.7 ± 1.1	12.57
MnO	0.200 ± 0	0.194 ± 0	0.193 ± 0	0.196 ± 0.008	Sr	310.2 ± 5.6	319.1 ± 4.1	320.2 ± 1.4	346 ± 14
MgO	3.55 ± 0.02	3.54 ± 0.01	3.56 ± 0.01	3.59 ± 0.05	V	419.6 ± 2.6	404.3 ± 5.8	409.5 ± 5.4	416 ± 14
CaO	7.14 ± 0	7.13 ± 0.02	7.12 ± 0.01	7.12 ± 0.11	Zr	171.0 ± 2.6	165.1 ± 0.3	155.3 ± 0.9	188 ± 16
Na_2O	3.16 ± 0.04	3.17 ± 0.02	3.17 ± 0.01	3.16 ± 0.11	Zn	121.9 ±6.3	134.7 ± 3.0	134.2 ± 2.7	127 ± 9
K_2O	1.75 ± 0	1.75 ± 0	1.75 ± 0	1.79 ± 0.05					
P_2O_5	0.348 ± 0.001	0.359 ± 0.001	0.348 ± 0.002	0.35 ± 0.02					
LOI	-0.06 ± 0.03	-0.06 ± 0.03	-0.06 ± 0.03	NRV					

Total	99.79	99.75	99.68	NC					
BHVO-2 (basalt)									
Major	This work			Literature	Minor	This work			Literature
	$X_{GR} \pm SD_{GR}$	$X_{ST} \pm SD_{ST}$	$X_{SH} \pm SD_{SH}$	$X_{lit} \pm SD_{lit}$		$X_{GR} \pm SD_{GR}$	$X_{ST} \pm SD_{ST}$	$X_{SH} \pm SD_{SH}$	$X_{lit} \pm SD_{lit}$
SiO_2	49.70 ± 0.15	49.75 ± 0.07	49.91 ± 0.11	49.90 ± 0.6	Cr	266.6 ± 5.6	275.2 ± 2.4	277.9 ± 2.7	280 ± 19
TiO_2	2.827 ± 0.005	2.785 ± 0.004	2.780 ± 0.007	2.73 ± 0.04	Cu	91.1 ± 3.2	100.9 ± 2.7	98.5 ± 4.1	127 ± 7
Al_2O_3	13.46 ± 0.02	13.46 ± 0.03	13.52 ± 0.01	13.50 ± 0.2	Ba	132.0 ± 23.2	103.6 ± 1.8	101.9 ± 11.6	130 ± 13
TFe_2O_3	12.37 ± 0.03	12.34 ± 0.01	12.37 ± 0.02	12.30 ± 0.2	Ni	110.6 ± 1.4	113.8 ± 2.3	114.2 ± 3.2	119 ± 7
MnO	0.170 ± 0	0.166 ± 0.001	0.165 ± 0	0.167 ± 0.005	Sr	359.4 ± 5.0	373.9 ± 5.6	377.0 ± 2.4	389 ± 23
MgO	7.13 ± 0.01	7.15 ± 0.02	7.15 ± 0	7.23 ± 0.12	V	337.2 ± 2.3	307.2 ± 5.2	303.9 ± 4.5	317 ± 11
CaO	11.35 ± 0.03	11.35 ± 0.03	11.37 ± 0.03	11.40 ± 0.2	Zr	158.6 ± 0.6	153.6 ± 1.0	133.3 ± 0.6	172 ± 11
Na_2O	2.22 ± 0.02	2.22 ± 0.01	2.23 ± 0.01	2.22 ± 0.08	Zn	86.5 ± 2.2	97.0 ± 1.5	99.8 ± 1.6	103 ± 6
K_2O	0.51 ± 0	0.50 ± 0	0.51 ± 0	0.52 ± 0.01					
P_2O_5	0.262 ± 0.001	0.270 ± 0.002	0.262 ± 0.002	0.27 ± 0.02					
LOI	-0.55 ± 0.03	-0.55 ± 0.03	-0.55 ± 0.03	NRV					
Total	99.45	99.42	99.72	NC					
W-2a (diabase)									
Major	This work			Literature	Minor	This work			Literature
	$X_{GR} \pm SD_{GR}$	$X_{ST} \pm SD_{ST}$	$X_{SH} \pm SD_{SH}$	$X_{lit} \pm SD_{lit}$		$X_{GR} \pm SD_{GR}$	$X_{ST} \pm SD_{ST}$	$X_{SH} \pm SD_{SH}$	$X_{lit} \pm SD_{lit}$
SiO_2	52.30 ± 0.05	52.28 ± 0.06	52.33 ± 0.08	52.44 ± 0.64	Cr	87.8 ± 10.2	89.1 ± 2.9	92.8 ± 2.4	93 ± 6
TiO_2	1.026 ± 0.018	1.049 ± 0.001	1.056 ± 0.002	1.06 ± 0.03	Cu	78.0 ± 4.4	87.5 ± 2.1	88.1 ± 0.9	103 ± 12
Al_2O_3	15.21 ± 0.02	15.2 ± 0.01	15.32 ± 0.04	15.35 ± 0.23	Ba	182.8 ± 14.8	174.9 ± 12.6	170.8 ± 8.6	182 ± 23
TFe_2O_3	10.79 ± 0.04	10.74 ± 0.03	10.73 ± 0.01	10.74 ± 0.23	Ni	64.5 ± 7.4	67.1 ± 3.1	66.6 ± 3.2	70 ± 8
MnO	0.170 ± 0	0.164 ± 0.001	0.163 ± 0.001	0.163 ± 0.009	Sr	163.9 ± 1.4	172.2 ± 2.9	170.3 ± 2.9	194 ± 17
MgO	6.28 ± 0.01	6.27 ± 0.01	6.29 ± 0.01	6.37 ± 0.18	V	251.7 ± 6.3	258.2 ± 2.6	264.9 ± 1.6	262 ± 14
CaO	10.81 ± 0.02	10.78 ± 0.01	10.78 ± 0.02	10.87 ± 0.29	Zr	95.3 ± 1.7	91.1 ± 0.6	77.8 ± 0.3	94 ± 9
Na_2O	2.22 ± 0.01	2.22 ± 0	2.23 ± 0.01	2.14 ± 0.12	Zn	65.1 ± 7.5	72.7 ± 3.3	72.6 ± 1.6	77 ± 5
K_2O	0.620 ± 0	0.613 ± 0	0.618 ± 0	0.627 ± 0.025					
P_2O_5	0.123 ± 0.001	0.127 ± 0.001	0.124 ± 0.001	0.131 ± 0.016					
LOI	-0.01 ± 0.08	-0.01 ± 0.08	-0.01 ± 0.08	NRV					

Total	99.52	99.43	99.62	NC					
RGM-2 (rhyolite)									
Major	This work			Literature	Minor	This work			Literature
	$X_{GR} \pm SD_{GR}$	$X_{ST} \pm SD_{ST}$	$X_{SH} \pm SD_{SH}$	$X_{lit} \pm SD_{lit}$		$X_{GR} \pm SD_{GR}$	$X_{ST} \pm SD_{ST}$	$X_{SH} \pm SD_{SH}$	$X_{lit} \pm SD_{lit}$
SiO_2	73.32 ± 0.17	73.17 ± 0.04	73.40 ± 0.03	73.40 ± 0.41	Cr	*10.8 ± 16.8*	*5.2 ± 5.2*	*5.7 ± 2.4*	4
TiO_2	0.266 ± 0.005	0.272 ± 0.003	0.273 ± 0.001	0.25 ± 0.023	Cu	*10.5 ± 2.4*	*26.1 ± 2.1*	*25.9 ± 3.3*	9.8 ± 0.8
Al_2O_3	13.64 ± 0.06	13.61 ± 0.02	13.67 ± 0.02	14.00 ± 0.3	Ba	837.0 ± 4.3	853.2 ±20.8	801.9 ± 24.8	842 ± 35
TFe_2O_3	1.84 ± 0.01	1.84 ± 0.01	1.84 ± 0.01	1.86 ± 0.04	Ni	*15.5 ± 37.8*	*1.2 ± 2.2*	*1.4 ± 2.8*	4
MnO	0.030 ± 0	0.035 ± 0.001	0.035 ± 0.001	0.035 ± 0.001	Sr	87.6 ± 0.6	91.6 ± 0.8	99.3 ± 0.5	108 ± 5
MgO	0.29 ± 0.01	0.28 ± 0.01	0.29 ± 0.01	0.28 ± 0.02	V	13.1 ± 4.0	17.7 ± 11.0	15.9 ± 3.5	NRV
CaO	1.22 ± 0.01	1.23 ± 0.01	1.23 ± 0.01	1.23 ± 0.03	Zr	217.0 ± 1.7	215.8 ± 0.8	200.0 ± 0.8	222 ± 17
Na_2O	4.10 ± 0.03	4.11 ± 0.01	4.13 ± 0	4.14 ± 0.12	Zn	19.5 ± 2.5	30.0 ± 3.8	29.0 ± 2.8	33 ± 2
K_2O	4.31 ± 0.01	4.30 ± 0	4.32 ± 0.01	4.35 ± 0.16					
P_2O_5	0.049 ± 0.001	0.049 ± 0.001	0.050 ± 0	0.05 ± 0.014					
LOI	0.69 ± 0.04	0.69 ± 0.04	0.69 ± 0.04	NRV					
Total	99.74	99.59	99.92	NC					
AC-E (granite)									
Major	This work			Literature	Minor	This work			Literature
	$X_{GR} \pm SD_{GR}$	$X_{ST} \pm SD_{ST}$	$X_{SH} \pm SD_{SH}$	$X_{lit} \pm SD_{lit}$		$X_{GR} \pm SD_{GR}$	$X_{ST} \pm SD_{ST}$	$X_{SH} \pm SD_{SH}$	$X_{lit} \pm SD_{lit}$
SiO_2	70.63 ± 0.07	70.61 ± 0.14	70.49 ± 0.14	70.35 ± 0.42	Cr	*-4.9 ± 0.7*	*2.8 ± 1.8*	*5.7 ± 6.4*	3.4 ± 4.1
TiO_2	0.108 ± 0.002	0.105 ± 0.002	0.106 ± 0	0.11 ± 0.11	Cu	*8.8 ± 7.2*	*15.7 ± 2.4*	*14.1 ± 2.9*	4 ± 3.2
Al_2O_3	14.69 ± 0.05	14.65 ± 0.01	14.68 ± 0.04	14.70 ± 0.29	Ba	*71.5 ± 15.4*	*50.1 ± 18.9*	*55.8 ± 7.7*	55 ± 20
TFe_2O_3	2.49 ± 0.01	2.46 ± 0.01	2.48 ± 0.03	2.53 ± 0.13	Ni	*-1.9 ± 6.2*	*-4.0 ± 3.2*	*-2.6 ± 3.5*	1.5 ± 4.4
MnO	0.060 ± 0	0.058 ± 0.001	0.057 ± 0.001	0.058 ± 0.010	Sr	*-43.6 ± 0.3*	*-42.3 ± 1.4*	*-33.6 ± 1.4*	3 ± 3
MgO	0.03 ± 0.01	0.03 ± 0	0.03 ± 0	0.03 ± 0.04	V	*2.7 ± 5.0*	*-2.5 ± 2.6*	*-3.3 ± 7.7*	3 ± 2.4
CaO	0.35 ± 0.01	0.36 ± 0.01	0.35 ± 0	0.34 ± 0.09	Zr	789.5 ± 8.6	792.8 ± 4.5	728.1 ± 7.8	780 ± 81
Na_2O	6.50 ± 0.08	6.58 ± 0.02	6.59 ± 0.02	6.54 ± 0.23	Zn	296.9 ± 10.1	313.4 ± 2.5	311.2 ± 1.6	224 ± 26
K_2O	4.44 ± 0.01	4.46 ± 0.01	4.46 ± 0.01	4.49 ± 0.13					
P_2O_5	0.015 ± 0.001	0.013 ± 0.001	0.016 ± 0	0.014 ± 0.020					
LOI	0.27 ± 0.09	0.27 ± 0.09	0.27 ± 0.09	0.37 ± 0.12					

Total	99.57	99.59	99.53	99.53					
GH (granite)									
Major	This work			Literature	Minor	This work			Literature
	$X_{GR} \pm SD_{GR}$	$X_{ST} \pm SD_{ST}$	$X_{SH} \pm SD_{SH}$	$X_{lit} \pm SD_{lit}$		$X_{GR} \pm SD_{GR}$	$X_{ST} \pm SD_{ST}$	$X_{SH} \pm SD_{SH}$	$X_{lit} \pm SD_{lit}$
SiO_2	75.73 ± 0.14	75.69 ± 0.08	75.68 ± 0.05	75.80 ± 0.61	Cr	*0.8 ± 3.3*	*3.9 ± 2.8*	*-0.8 ± 5.2*	3 ± 7.4
TiO_2	0.080 ± 0	0.077 ± 0.001	0.077 ± 0.003	0.08 ± 0.04	Cu	*6.9 ± 3.7*	*15.7 ± 1.6*	*17.1 ± 2.3*	3 ± 7.2
Al_2O_3	12.48 ± 0.06	12.50 ± 0.03	12.51 ± 0.01	12.50 ± 0.35	Ba	*11.3 ± 9.4*	*27.4 ± 8.0*	*22.7 ± 15.8*	20 ± 32.9
TFe_2O_3	1.32 ± 0.01	1.3 ± 0	1.31 ± 0	1.34 ± 0.23	Ni	*-1.0 ± 1.8*	*-2.5 ± 2.7*	*1.1 ± 1.4*	3 ± 4.8
MnO	0.050 ± 0	0.046 ± 0.001	0.046 ± 0.001	0.05 ± 0.01	Sr	*-37.8 ± 0.6*	*-34.3 ± 2.3*	*-22.4 ± 2.1*	10
MgO	0.04 ± 0.01	0.03 ± 0.01	0.04 ± 0	0.03 ± 0.1	V	*0.2 ± 7.1*	*-0.5 ± 3.4*	*3.3 ± 8.5*	5 ± 7.8
CaO	0.72 ± 0.01	0.73 ± 0	0.73 ± 0	0.69 ± 0.13	Zr	151.7 ± 0.7	151.4 ± 0.6	127.0 ± 2.3	150 ± 65.7
Na_2O	3.78 ± 0.05	3.80 ± 0	3.82 ± 0.01	3.85 ± 0.22	Zn	52.3 ± 3.5	59.4 ± 3.4	60.4 ± 4.3	55 ± 17.4
K_2O	4.72 ± 0	4.74 ± 0.01	4.73 ± 0.01	4.76 ± 0.15					
P_2O_5	0.010 ± 0.002	0.007 ± 0.001	0.011 ± 0.001	0.01 ± 0.01					
LOI	0.77 ± 0.05	0.77 ± 0.05	0.77 ± 0.05	0.70					
Total	99.71	99.69	99.72	99.81					
PM-S (microgabbro)									
Major	This work			Literature	Minor	This work			Literature
	$X_{GR} \pm SD_{GR}$	$X_{ST} \pm SD_{ST}$	$X_{SH} \pm SD_{SH}$	$X_{lit} \pm SD_{lit}$		$X_{GR} \pm SD_{GR}$	$X_{ST} \pm SD_{ST}$	$X_{SH} \pm SD_{SH}$	$X_{lit} \pm SD_{lit}$
SiO_2	47.00 ± 0.05	46.90 ± 0.02	46.78 ± 0.22	47.00 ± 0.65	Cr	292.8 ± 4.4	292.9 ± 4.1	299.7 ± 3.7	314 ± 26.9
TiO_2	1.065 ± 0.025	1.094 ± 0.003	1.096 ± 0.005	1.10 ± 0.05	Cu	43.3 ± 3.5	49.9 ± 4.8	51.0 ± 1.9	59 ± 7.33
Al_2O_3	17.06 ± 0.03	17.06 ± 0.02	16.97 ± 0.07	17.15 ± 0.48	Ba	151.8 ± 0.6	155.8 ± 16.6	147.9 ± 6.2	148 ± 14.95
TFe_2O_3	10.02 ± 0.01	10.00 ± 0.01	9.95 ± 0.05	10.1 ± 0.28	Ni	110.1 ± 5.7	116.5 ± 2.0	112.9 ± 1.6	115 ± 11.83
MnO	0.160 ± 0	0.154 ± 0.001	0.152 ± 0.001	0.16 ± 0.01	Sr	252.5 ± 0.7	261.3 ± 1.7	257.3 ± 2.1	280 ± 23.15
MgO	9.22 ± 0.03	9.27 ± 0.01	9.27 ± 0.05	9.34 ± 0.39	V	176.4 ± 4.5	181.5 ± 3.8	183.1 ± 1.1	192 ± 22.49
CaO	12.22 ± 0.02	12.17 ± 0.01	12.12 ± 0.06	12.48 ± 0.26	Zr	43.1 ± 0.5	40.0 ± 2.0	46.1 ± 0.7	39 ± 7.2
Na_2O	2.03 ± 0.02	2.03 ± 0	2.03 ± 0.02	2.08 ± 0.11	Zn	47.0 ± 7.6	49.4 ± 2.2	51.1 ± 2.0	60 ± 6.74
K_2O	0.14 ± 0	0.13 ± 0	0.14 ± 0	0.14 ± 0.02					
P_2O_5	0.032 ± 0.001	0.029 ± 0.001	0.032 ± 0.001	0.03 ± 0.05					
LOI	0.38 ± 0.12	0.38 ± 0.12	0.38 ± 0.12	0.3					

Total	99.33	99.22	98.91	99.88					
WS-E (dolerite)									
Major	This work			Literature	Minor	This work			Literature
	$X_{GR} \pm SD_{GR}$	$X_{ST} \pm SD_{ST}$	$X_{SH} \pm SD_{SH}$	$X_{lit} \pm SD_{lit}$		$X_{GR} \pm SD_{GR}$	$X_{ST} \pm SD_{ST}$	$X_{SH} \pm SD_{SH}$	$X_{lit} \pm SD_{lit}$
SiO_2	51.13 ± 0.03	50.93 ± 0.01	51.15 ± 0.22	50.70 ± 0.58	Cr	82.6 ± 3.4	95.6 ± 4.0	96.3 ± 1.7	99 ± 9.9
TiO_2	2.506 ± 0.003	2.474 ± 0.006	2.453 ± 0.011	2.40 ± 0.08	Cu	41.8 ± 4.3	50.8 ± 2.3	51.8 ± 2.9	65 ± 10.84
Al_2O_3	13.73 ± 0.04	13.64 ± 0.01	13.69 ± 0.07	13.78 ± 0.28	Ba	324.1 ± 27.4	316.3 ± 10.0	333.9 ± 11.2	338 ± 27.52
TFe_2O_3	13.23 ± 0.02	13.2 ± 0.02	13.2 ± 0.07	13.15 ± 0.37	Ni	50.4 ± 1.5	50.2 ± 3.4	57.2 ± 8.3	55 ± 7.51
MnO	0.170 ± 0	0.168 ± 0.001	0.167 ± 0.001	0.17 ± 0.01	Sr	384.6 ± 1.6	395.4 ± 1.5	396.4 ± 3.8	410 ± 25.62
MgO	5.51 ± 0.02	5.48 ± 0.02	5.50 ± 0.03	5.55 ± 0.21	V	345.7 ± 4.7	326.3 ± 7.5	327.6 ± 6.4	340 ± 31.83
CaO	9.00 ± 0	8.96 ± 0.01	8.96 ± 0.04	8.95 ± 0.25	Zr	189.3 ± 1.2	184.8 ± 1.1	166.8 ± 0.5	195 ± 10.92
Na_2O	2.48 ± 0.04	2.45 ± 0.01	2.47 ± 0.01	2.47 ± 0.14	Zn	101.8 ± 1.1	113.5 ± 1.7	114.1 ± 2.0	117 ± 0.22
K_2O	0.99 ± 0	0.97 ± 0	0.98 ± 0	1.00 ± 0.06					
P_2O_5	0.296 ± 0.002	0.303 ± 0.002	0.298 ± 0.003	0.30 ± 0.04					
LOI	0.88 ± 0.05	0.88 ± 0.05	0.88 ± 0.05	0.85					
Total	99.92	99.46	99.75	99.32					
AN-G (anorthosite)									
Major	This work			Literature	Minor	This work			Literature
	$X_{GR} \pm SD_{GR}$	$X_{ST} \pm SD_{ST}$	$X_{SH} \pm SD_{SH}$	$X_{lit} \pm SD_{lit}$		$X_{GR} \pm SD_{GR}$	$X_{ST} \pm SD_{ST}$	$X_{SH} \pm SD_{SH}$	$X_{lit} \pm SD_{lit}$
SiO_2	46.11 ± 0.07	46.04 ± 0.08	46.68 ± 0.11	46.30 ± 0.69	Cr	45.3 ± 5.7	40.0 ± 1.1	48.9 ± 5.1	50 ± 10.4
TiO_2	0.219 ± 0.004	0.217 ± 0.003	0.219 ± 0.002	0.22 ± 0.04	Cu	11.8 ± 2.4	23.9 ± 2.4	23.8 ± 2.7	19 ± 4.58
Al_2O_3	29.89 ± 0.05	29.88 ± 0.05	29.68 ± 0.10	29.80 ± 0.94	Ba	*15.4 ± 9.0*	*17.2 ± 7.8*	*32.5 ± 19.3*	34 ± 11
TFe_2O_3	3.23 ± 0.01	3.22 ± 0	3.22 ± 0.01	3.36 ± 0.20	Ni	17.0 ± 1.2	25.6 ± 1.4	27.2 ± 1.4	35 ± 9.75
MnO	0.040 ± 0	0.044 ± 0.001	0.043 ± 0	0.04 ± 0.01	Sr	45.6 ± 0.3	49.2 ± 1.2	49.2 ± 2.2	76 ± 14.84
MgO	1.68 ± 0.01	1.77 ± 0.01	1.77 ± 0.01	1.80 ± 0.18	V	65.0 ± 2.6	70.6 ± 5.7	65.3 ± 4.7	70 ± 13.64
CaO	15.55 ± 0.02	15.51 ± 0.02	15.40 ± 0.03	15.90 ± 0.50	Zr	23.2 ± 0.9	20.2 ± 1.3	19.2 ± 0.4	11 ± 7.68
Na_2O	1.65 ± 0.03	1.64 ± 0	1.65 ± 0.01	1.63 ± 0.17	Zn	6.3 ± 5.8	5.9 ± 1.7	6.1 ± 2.5	20 ± 11.58
K_2O	0.14 ± 0	0.13 ± 0	0.14 ± 0	0.13 ± 0.04					
P_2O_5	0.016 ± 0	0.014 ± 0.001	0.018 ± 0	0.01 ± 0.06					
LOI	0.63 ± 0.02	0.63 ± 0.02	0.63 ± 0.02	0.65 ± 0.66					

Total	99.15	99.09	99.44	99.84					
MGL-OShBO (granite)									
Major	This work			Literature	Minor	This work			Literature
	$X_{GR} \pm SD_{GR}$	$X_{ST} \pm SD_{ST}$	$X_{SH} \pm SD_{SH}$	$X_{lit} \pm SD_{lit}$		$X_{GR} \pm SD_{GR}$	$X_{ST} \pm SD_{ST}$	$X_{SH} \pm SD_{SH}$	$X_{lit} \pm SD_{lit}$
SiO_2	71.98 ± 0.24	71.59 ± 0.12	71.82 ± 0.08	71.72 ± 0.572	Cr	179.1 ± 2.3	190.6 ± 6.8	188.1 ± 6.0	164 ± 38.1
TiO_2	0.014 ± 0.001	0.010 ± 0.001	0.010 ± 0.001	NRV	Cu	*13.5 ± 2.2*	*21.9 ± 3.7*	*21.9 ± 1.0*	7.1 ± 1.17
Al_2O_3	15.96 ± 0.07	15.97 ± 0.04	16.02 ± 0.03	16.12 ± 0.362	Ba	*6.6 ± 27.7*	*23.1 ± 10.9*	*1.2 ± 16.9*	12.3 ± 0.79
TFe_2O_3	0.51 ± 0	0.39 ± 0.01	0.41 ± 0.01	0.500 ± 0.032	Ni	10.4 ± 4.1	13.2 ± 1.7	14.4 ± 1.2	10.7 ± 2.91
MnO	0.160 ± 0	0.155 ± 0	0.155 ± 0.001	0.149 ± 0.014	Sr	*-32.3 ± 1.0*	*-32.0 ± 0.8*	*-17.1 ± 3.1*	12.3 ± 2.02
MgO	0.02 ± 0.01	0.03 ± 0.01	0.03 ± 0	NRV	V	*2.7 ± 3.0*	*2.5 ± 1.6*	*3.1 ± 4.3*	NRV
CaO	0.39 ± 0.01	0.39 ± 0	0.39 ± 0	0.388 ± 0.019	Zr	31.8 ± 1.1	32.2 ± 0.8	23.2 ± 0.5	40.1 ± 3.41
Na_2O	5.43 ± 0.05	5.44 ± 0.01	5.46 ± 0.03	5.34 ± 0.189	Zn	110.0 ± 2.9	120.3 ± 3.8	125.1 ± 1.7	92.4 ± 7.59
K_2O	3.56 ± 0	3.55 ± 0.01	3.57 ± 0.01	3.58 ± 0.105					
P_2O_5	0.024 ± 0.001	0.024 ± 0	0.025 ± 0.001	0.029 ± 0.002					
LOI	1.09 ± 0.04	1.09 ± 0.04	1.09 ± 0.04	1.10 ± 0.066					
Total	99.13	98.63	98.98	NC					

The symbols used in the table are explained as follows: X_{GR} is the mean measured value (n = 3) and SD_{GR} is the corresponding standard deviation value for the grinding (GR) method; X_{ST} is the mean measured value (n = 3) and SD_{ST} is the corresponding standard deviation value for the stirring rod (ST) method; X_{SH} is the mean measured value (n = 3) and SD_{SH} is the corresponding standard deviation value for the shaker cup (SH) method; X_{lit} is the recommended value and SD_{lit} is the corresponding recommended standard deviation value by literature; NRV = no recommended value; NC = no calculation. The ST and SH methods adopt the LOI values of the GR method. Some erroneous data are marked in italics and red.

Table S3. Source of recommend values of 19 CRMs.

Certificated reference materials	Source of recommend values
JG-2, JG-1a, JA-3, JR-2	Shigeru et al. [1]
	Noboru IMAI et al. [2]
JB-2a	Certified Geochemical Reference Material GSJ CRM JB-2a Basalt (Oshima volcano) Geochemical Reference Material Technical Information
JB-3a	Certified Geochemical Reference Material GSJ CRM JB-3a Basalt (Fuji volcano) Geochemical Reference Material Technical Information
JH-1, JGb-2	Imai et al. [3]
GSP-2	Wilson S.A. [4]
BCR-2	Wilson S.A. [5]
BHVO-2	Wilson S.A. [6]
W-2a	Gladney et al. [7]
RGM-2	United States Geological Survey Certificate of Analysis
AC-E, GH, PM-S, WS-E, AN-G	K. Govindaraju [8]
MGL-OShBO	Jean et al. [9]

Table S4. The values of $J_{S;i}$ by grinding method.

Samples	$J_{S;i}$ (GR)																	
	Major oxides										Minor elements							
	SiO_2	TiO_2	Al_2O_3	TFe_2O_3	MnO	MgO	CaO	Na_2O	K_2O	P_2O_5	Cr	Cu	Ba	Ni	Sr	V	Zr	Zn
JG-2	0.07	0.58	0.01	0.03	1.78	1.42	0.04	0.03	0.05	NC	NC	NC	0.07	NC	NC	NC	0.33	NC
JG-1a	0.04	0.11	0.13	0.16	0.18	0.00	0.06	0.02	0.00	0.00	0.46	NC	0.04	NC	0.11	0.06	0.03	2.84
JA-3	0.87	0.66	0.08	0.30	0.01	0.42	0.32	0.07	0.43	0.04	0.01	0.00	0.00	0.14	0.12	0.43	0.99	0.02
JB-2a	NC	NC	NC	NC	NC	NC	NC	NC	NC	NC	NC	NC	NC	NC	NC	NC	NC	NC
JB-3a	NC	NC	NC	NC	NC	NC	NC	NC	NC	NC	NC	NC	NC	NC	NC	NC	NC	NC
JH-1	0.15	0.63	0.01	0.34	1.42	0.15	3.72	0.04	0.08	0.01	0.11	NC	1.42	0.02	21.24	0.73	0.70	0.05
JGb-2	0.24	0.60	0.01	0.01	0.16	1.17	9.53	0.16	0.02	0.06	0.83	14.52	NC	0.12	1.05	0.93	0.05	6.38
JR-2	1.05	0.15	0.41	0.00	0.08	2.00	0.11	0.08	0.01	0.00	NC	NC	NC	NC	NC	NC	0.29	6.31
GSP-2	0.06	0.70	0.47	0.03	0.11	0.10	0.10	0.01	0.02	0.20	1.79	0.00	0.04	5.58	0.10	1.09	0.46	0.14
BCR-2	0.01	2.41	0.41	0.01	0.25	0.55	0.03	0.00	0.64	0.01	2.43	38.09	0.35	NC	5.64	0.06	1.10	0.22
BHVO-2	0.10	5.79	0.04	0.12	0.36	0.69	0.06	0.00	1.00	0.16	0.46	21.76	0.01	1.38	1.58	3.23	1.48	6.67
W-2a	1.67	2.73	2.22	0.04	0.56	2.34	0.39	0.27	0.25	0.02	0.19	3.83	0.00	0.25	3.11	0.45	0.02	1.74
RGM-2	0.03	0.46	1.38	0.24	25.00	0.20	0.10	0.10	0.06	0.01	NC	NC	0.02	NC	16.41	NC	0.09	17.78
AC-E	0.43	0.00	0.00	0.09	0.04	0.00	0.01	0.03	0.15	0.00	NC	NC	0.43	NC	NC	NC	0.01	6.83
GH	0.01	0.00	0.00	0.01	0.00	0.01	0.05	0.10	0.07	0.00	NC	NC	NC	NC	NC	NC	0.00	0.02
PM-S	0.00	0.39	0.04	0.08	0.00	0.09	0.99	0.20	0.00	0.00	0.60	3.74	0.06	0.14	1.41	0.46	0.32	1.64
WS-E	0.55	1.75	0.03	0.05	0.00	0.04	0.04	0.00	0.03	0.01	2.45	3.96	0.13	0.36	0.98	0.03	0.27	183.60
AN-G	0.08	0.00	0.01	0.42	0.00	0.44	0.49	0.01	0.06	0.01	0.16	1.94	NC	3.36	4.19	0.13	2.49	1.12
MGL-OShBO	0.18	NC	0.19	0.10	0.62	NC	0.01	0.21	0.04	7.78	0.16	NC	NC	0.00	NC	NC	5.37	4.69

NC=no calculation.

Table S5. The values of $J_{S;i}$ by stirring rod method.

Samples	$J_{S;i}$(ST)																	
	Major oxides										Minor elements							
	SiO_2	TiO_2	Al_2O_3	TFe_2O_3	MnO	MgO	CaO	Na_2O	K_2O	P_2O_5	Cr	Cu	Ba	Ni	Sr	V	Zr	Zn
JG-2	0.09	0.11	0.05	0.00	1.00	1.17	0.00	0.07	0.11	NC	NC	NC	0.01	NC	NC	NC	0.52	NC
JG-1a	0.01	0.05	0.29	0.25	0.08	0.02	0.14	0.02	0.00	0.00	0.26	NC	0.00	NC	0.00	0.00	0.01	0.19
JA-3	0.42	0.28	0.19	0.37	0.01	0.30	0.39	0.01	0.44	0.00	0.01	0.10	0.27	1.17	0.15	0.28	1.26	0.00
JB-2a	NC	NC	NC	NC	NC	NC	NC	NC	NC	NC	NC	NC	NC	NC	NC	NC	NC	NC
JB-3a	NC	NC	NC	NC	NC	NC	NC	NC	NC	NC	NC	NC	NC	NC	NC	NC	NC	NC
JH-1	0.28	0.16	0.00	1.20	2.40	0.16	5.75	0.40	0.04	0.01	0.16	NC	0.02	0.65	16.87	0.13	0.18	2.45
JGb-2	0.86	0.31	0.01	0.27	0.09	1.00	14.16	0.00	0.17	0.39	0.79	1.06	NC	1.03	2.79	0.65	0.01	1.59
JR-2	0.81	0.45	0.50	1.73	0.51	1.00	0.24	0.00	0.00	0.62	NC	NC	NC	NC	NC	NC	0.26	4.68
GSP-2	0.06	0.00	0.46	0.04	0.00	0.00	0.00	0.01	0.00	0.00	0.01	2.74	0.03	2.44	0.52	0.84	0.14	2.46
BCR-2	0.01	0.99	0.24	0.01	0.06	0.96	0.01	0.01	0.64	0.20	0.00	1.24	0.01	NC	3.40	0.60	2.05	0.66
BHVO-2	0.06	1.87	0.04	0.04	0.04	0.43	0.06	0.00	4.00	0.00	0.06	12.10	4.05	0.50	0.41	0.65	2.78	0.94
W-2a	1.82	1.20	2.43	0.18	0.53	2.89	1.03	0.29	1.17	0.01	0.34	1.62	0.07	0.11	1.60	0.07	0.10	0.52
RGM-2	0.31	0.90	1.68	0.24	0.00	0.00	0.00	0.06	0.10	0.01	NC	NC	0.08	NC	10.49	NC	0.13	0.49
AC-E	0.34	0.00	0.03	0.29	0.00	0.00	0.05	0.03	0.05	0.00	NC	NC	0.03	NC	NC	NC	0.02	11.71
GH	0.03	0.01	0.00	0.03	0.16	0.00	0.09	0.05	0.02	0.09	NC	NC	NC	NC	NC	NC	0.00	0.06
PM-S	0.02	0.01	0.04	0.13	0.36	0.03	1.42	0.21	0.25	0.00	0.60	1.08	0.12	0.02	0.65	0.21	0.02	2.24
WS-E	0.16	0.85	0.25	0.02	0.04	0.11	0.00	0.02	0.25	0.01	0.10	1.64	0.55	0.34	0.32	0.18	0.86	4.17
AN-G	0.14	0.01	0.01	0.49	0.16	0.03	0.61	0.00	0.00	0.00	0.91	0.90	NC	0.91	3.24	0.00	1.40	1.45
MGL-OShBO	0.05	NC	0.17	10.77	0.18		0.01	0.28	0.08	10.09	0.47	NC	NC	0.55	NC	NC	5.09	10.80

NC=no calculation.

Table S6. The values of $J_{S;i}$ by shaker cup method.

Samples	$J_{S;i}$ (SH)																
	Major oxides									Minor elements							
	SiO_2	TiO_2	Al_2O_3	TFe_2O_3	MnO	MgO	CaO	Na_2O	K_2O	P_2O_5	Cr	Cu	Ba	Ni	Sr	V	Zr	Zn
JG-2	0.11	0.20	0.05	0.09	0.44	1.17	0.00	0.01	0.01	NC	NC	NC	1.08	NC	NC	NC	0.37	NC
JG-1a	0.08	0.04	0.17	0.36	0.02	0.02	0.14	0.02	0.02	0.01	0.08	NC	0.15	NC	0.49	0.78	0.00	0.71
JA-3	0.24	0.17	0.42	0.37	0.01	0.11	0.38	0.00	0.25	0.01	0.02	0.17	0.01	0.41	0.32	0.00	1.74	0.22
JB-2a	NC	NC	NC	NC	NC	NC	NC	NC	NC	NC	NC	NC	NC	NC	NC	NC	NC	NC
JB-3a	NC	NC	NC	NC	NC	NC	NC	NC	NC	NC	NC	NC	NC	NC	NC	NC	NC	NC
JH-1	0.15	0.03	0.01	0.83	2.40	0.06	4.87	0.06	0.04	0.01	0.09	NC	2.67	0.47	25.63	0.80	1.50	0.59
JGb-2	0.57	0.02	0.00	0.14	0.09	0.37	12.15	1.01	0.17	0.02	2.65	2.04	NC	1.58	4.00	0.03	23.94	1.27
JR-2	0.11	0.49	0.15	1.00	0.50	0.50	0.11	0.00	0.01	0.00	NC	NC	NC	NC	NC	NC	6.13	3.19
GSP-2	0.07	0.06	0.00	0.02	0.11	0.10	0.00	0.11	0.02	0.06	0.40	1.79	0.09	0.48	1.14	1.49	3.24	3.04
BCR-2	0.01	0.43	0.30	0.04	0.14	0.35	0.00	0.01	0.64	0.01	0.00	2.91	0.01	NC	3.36	0.19	4.16	0.59
BHVO-2	0.00	1.52	0.01	0.12	0.16	0.44	0.02	0.02	1.00	0.16	0.01	12.34	2.60	0.39	0.27	1.21	12.34	0.27
W-2a	1.35	0.15	0.62	0.23	0.94	1.85	0.99	0.61	0.44	0.02	0.00	1.53	0.21	0.16	1.89	0.04	3.24	0.70
RGM-2	0.00	1.00	1.20	0.24	0.00	0.20	0.00	0.01	0.04	0.00	NC	NC	0.87	NC	3.00	NC	1.67	1.35
AC-E	0.10	0.00	0.00	0.14	0.01	0.00	0.01	0.05	0.05	0.01	NC	NC	0.00	NC	NC	NC	0.41	11.21
GH	0.04	0.01	0.00	0.02	0.16	0.01	0.09	0.02	0.04	0.01	NC	NC	NC	NC	NC	NC	0.12	0.09
PM-S	0.10	0.01	0.14	0.28	0.63	0.03	1.82	0.20	0.00	0.00	0.28	1.12	0.00	0.03	0.95	0.16	0.96	1.60
WS-E	0.53	0.43	0.10	0.02	0.09	0.06	0.00	0.00	0.11	0.00	0.07	1.38	0.02	0.04	0.28	0.15	6.65	2.08
AN-G	0.79	0.00	0.02	0.49	0.09	0.03	1.00	0.01	0.06	0.02	0.01	0.82	NC	0.63	3.19	0.11	1.14	1.38
MGL-OShBO	0.03	NC	0.08	7.21	0.18	NC	0.01	0.39	0.01	3.91	0.39	NC	NC	1.38	NC	NC	24.05	17.67

NC=no calculation.

References:

1. Shigeru TERASHIMA, N.I., Shiro ITOH, Atsushi ANDO and Naoki MITA. 1993 compilation of analytical data for major elements in seventeen GSJ geochemical reference samples, "Igneous rock series". *Bulletin of the Geological Survey of Japan* **1994**, *45*, 305-381.
2. IMAI, N.; TERASHIMA, S.; ITOH, S.; ANDO, A. 1994 compliation of analytical data for minor and trace elements in seventeen GSJ geochemical reference samples, "igneous rock series". *Geostandards Newsletter* **1995**, *19*, 135-213, doi:10.1111/j.1751-908X.1995.tb00158.x.
3. Imai, N.; Terashima, S.; Itoh, S.; Ando, A. 1998 Compilation of Analytical Data for Five GSJ Geochemical Reference Samples: The "Instrumental Analysis Series". *Geostandards Newsletter* **1999**, *23*, 223-250, doi:10.1111/j.1751-908X.1999.tb00576.x.
4. Wilson, S.A. Data compilation for USGS reference material GSP-2, Granodiorite, Silver Plume, Colorado. U.S. Geological Survey Open-File Report (in progress). **1998**.
5. Wilson, S.A. The collection, preparation and testing of USGS reference material BCR-2, Columbia River, Basalt, U.S. Geological Survey Open-File Report 98-00x. **1997**.
6. Wilson, S.A. Data compilation for USGS reference material BHVO-2, Hawaiian Basalt, U.S. Geological Survey Open-File Report xxxxx. **1997**.
7. GLADNEY, E.S.; ROELANDTS, I. 1987 Compilation of Elemental Concentration Data for USGS BIR-1, DNC-1 and W-2. *Geostandards Newsletter* **1988**, *12*, 63-118, doi:10.1111/j.1751-908X.1988.tb00044.x.
8. K.Govindaraju. 1995 working values with confidence limits for twenty-six CRPG, ANRT and IWG-GIT geostandards. *Geostandards Newsletter* **1995**, *19*, 1-32, doi:10.1111/j.1751-908X.1995.tb00164.x.
9. Kane, J.S.; Batjargal, B.; Erdenetsetseg, D. Report on the 2007 Recertification of the Certified Reference Materials GAS (Serpentinite) and OShBO (Alkaline Granite). *Geostandards and Geoanalytical Research* **2009**, *33*, 295-308, doi:10.1111/j.1751-908X.2009.00002.x.
